# Predictors of BILAG-based outcomes in patients with SLE: Analysis from the Systemic Lupus International Collaborating Clinics (SLICC) Inception Cohort

**DOI:** 10.1177/09612033231183273

**Published:** 2023-07-18

**Authors:** Trixy David, Li Su, Yafeng Cheng, Caroline Gordon, Benjamin Parker, David Isenberg, John A Reynolds, Ian N Bruce, Systemic Lupus International Collaborating Clinics (SLICC) consortium and MASTERPLANS consortium, SLICC Consortium, John G. Hanly, Sang-Cheol Bae, Juanita Romero-Diaz, Jorge Sanchez-Guerrero, Sasha Bernatsky, Ann E. Clarke, Daniel J Wallace, Anisur Rahman, Joan T. Merrill, Paul R. Fortin, Dafna D. Gladman, Murray B. Urowitz, Michelle Petri, Ellen M. Ginzler, M.A. Dooley, Rosalind Ramsey-Goldman, Susan Manzi, Andreas Jonsen, Graciela S. Alarcón, Ronald F. van Vollenhoven, Cynthia Aranow, Meggan Mackay, Guillermo Ruiz-Irastorza, S. Sam Lim, Murat Inanc, Kenneth C. Kalunian, Soren Jacobsen, Christine A. Peschken, Diane L. Kamen, Anca Askanase, MASTERPLANS Consortium Members, Ian N Bruce, Katherine Payne, Mark Lunt, Niels Peek, Nophar Geifman, Sean Gavan, Gillian Armitt, Patrick Doherty, Jennifer Prattley, Narges Azadbakht, Angela Papazian, Helen Le Sueur, Carmen Farrelly, Clare Richardson, Zunnaira Shabbir, Lauren Hewitt, Neil McHugh, Caroline Gordon, John Reynolds, Stephen Young, David Jayne, Vern Farewell, Li Su, Matthew Pickering, Elizabeth Lightstone, Alyssa Gilmore, Marina Botto, Timothy Vyse, David Lester Morris, D D’Cruz, Edward Vital, Miriam Wittmann, Paul Emery, Michael Beresford, Christian Hedrich, Angela Midgley, Jenna Gritzfeld, Michael Ehrenstein, David Isenberg, Mariea Parvaz, Jane Dunnage, Jane Batchelor, E Holland, Pauline Upsall

**Affiliations:** Division of Rheumatology, Department of Medicine and Department of Pathology, Queen Elizabeth II Health Sciences Centre and Dalhousie University, Halifax, Nova Scotia, Canada; Department of Rheumatology, Hanyang University Hospital for Rheumatic Diseases, Seoul, Korea; Instituto Nacional de Ciencias Médicas y Nutrición, Mexico City, Mexico; Divisions of Rheumatology and Clinical Epidemiology, Department of Medicine, McGill University, Montreal, Quebec, Canada; Division of Rheumatology, Cumming School of Medicine, University of Calgary, Calgary, Alberta, Canada; Cedars-Sinai/David Geffen School of Medicine at UCLA, Los Angeles, CA, USA; Centre for Rheumatology, Department of Medicine, University College London, UK; Department of Clinical Pharmacology, Oklahoma Medical Research Foundation, Oklahoma City, OK, USA; Division of Rheumatology, Department of Medicine, CHU de Québec - Université Laval, Quebec City, Canada; Centre for Prognosis Studies in the Rheumatic Diseases, Toronto Western Hospital and University of Toronto, ON, Canada; Division of Rheumatology, Johns Hopkins University School of Medicine, Baltimore, MD, USA; Department of Medicine, SUNY Downstate Medical Centre, Brooklyn, NY, USA; Thurston Arthritis Research Centre, University of North Carolina, Chapel Hill, NC, USA; Northwestern University and Feinberg School of Medicine, Chicago, IL, USA; Lupus Centre of Excellence, Allegheny Health Network, Pittsburgh, PA, USA; Department of Clinical Sciences Lund, Rheumatology, Lund University, Lund, Sweden; Department of Medicine, University of Alabama at Birmingham, Birmingham, AL, USA; Department of Rheumatology and Clinical Immunology, Amsterdam University Medical Centre, Amsterdam, Holland; Feinstein Institute for Medical Research, Manhasset, NY, USA; Autoimmune Diseases Research Unit, Department of Internal Medicine, BioCruces Bizkaia Health Research Institute, Hospital Universitario Cruces, University of the Basque Country, Barakaldo, Spain; Emory University, Department of Medicine, Division of Rheumatology, Atlanta, Georgia, USA; Division of Rheumatology, Department of Internal Medicine, Istanbul Medical Faculty, Istanbul University, Istanbul, Turkey; UCSD School of Medicine, La Jolla, CA, USA; Copenhagen Lupus and Vasculitis Clinic, 4242, Rigshospitalet, Copenhagen University Hospital, Copenhagen, Denmark; University of Manitoba, Winnipeg, Manitoba, Canada; Medical University of South Carolina, Charleston, South Carolina, USA; Hospital for Joint Diseases, NYU, Seligman Centre for Advanced Therapeutics, New York NY; The University of Manchester; University of Bath; University of Birmingham; University of Cambridge; Imperial College London; King’s College London; University of Leeds; University of Liverpool; University College London; MASTERPLANS Patient and Public Involvement Group; 1 The Kellgren Centre for Rheumatology, Manchester University NHS Foundation Trust, Manchester Academic Health Science Centre, Manchester, UK; 2 MRC Biostatistics Unit, School of Clinical Medicine, University of Cambridge, Cambridge, UK; 3 Rheumatology Research Group, Institute of Inflammation and Ageing, University of Birmingham, Birmingham, UK; 4 Centre for Rheumatology, Division of Medicine, University College London, London, UK; 5 Rheumatology Department, City Hospital, Sandwell and West Birmingham NHS Trust, Birmingham, UK; 6 Centre for Musculoskeletal Research, The University of Manchester, Faculty of Biology Medicine and Health, Manchester Academic Health Science Centre, Manchester, UK

## Abstract

**Background:**

We aimed to identify factors associated with a significant reduction in SLE disease activity over 12 months assessed by the BILAG Index.

**Methods:**

In an international SLE cohort, we studied patients from their ‘inception enrolment’ visit. We also defined an ‘active disease’ cohort of patients who had active disease similar to that needed for enrolment into clinical trials. Outcomes at 12 months were; Major Clinical Response (MCR: reduction to classic BILAG C in all domains, steroid dose of ≤7.5mg and SLEDAI ≤4) and ‘Improvement’ (reduction to <=1B score in previously active organs; no new BILAG A/B; stable or reduced steroid dose; no increase in SLEDAI). Univariate and multivariate logistic regression with Least Absolute Shrinkage and Selection Operator (LASSO) and cross-validation in randomly split samples were used to build prediction models.

**Results:**

‘Inception enrolment’ (n=1492) and ‘active disease’ (n=924) patients were studied. Models for MCR performed well (ROC AUC =0.777 and 0.732 in the inception enrolment and active disease cohorts respectively). Models for Improvement performed poorly (ROC AUC = 0.574 in the active disease cohort). MCR in both cohorts was associated with antimalarial use and inversely associated with active disease at baseline (BILAG or SLEDAI) scores, BILAG haematological A/B scores, higher steroid dose and immunosuppressive use.

**Conclusion:**

Baseline predictors of response in SLE can help identify patients in clinic who are less likely to respond to standard therapy. They are also important as stratification factors when designing clinical trials in order to better standardize overall usual care response rates.

## Background

Systemic Lupus Erythematosus (SLE) is a complex, multisystem autoimmune disease, associated with significant morbidity and mortality which carries a high socio-economic burden^1–4^. Most therapies used are off-label and the efficacy of each is limited^5^. Several novel therapies are in development, however optimal and cost-effective positioning of these in the drug treatment pathway poses major challenges. Precision medicine aims to identify patient endotypes that respond particularly well to a specific therapy and will be a vital step forward in the era of novel targeted therapies. The natural history of SLE is however also important to consider. Studying patients who respond well to current standard of care (SOC) will help to identify common (public) markers of an overall good outcome. These need to be understood and accounted for when assessing treatment-specific (private) response markers.

MASTERPLANS is an MRC-funded Precision Medicine consortium aiming to identify predictors of treatment response in SLE. Our consortium employed a series of BILAG-based definitions of Improvement and Major Clinical Response (MCR) to provide consistent outcome assessments across cohorts and trial populations.

Our hypothesis is that there are certain factors associated with improvement in SLE disease activity over time, in the setting of ‘usual care’. Using data from the Systemic Lupus International Collaborating Clinics (SLICC) Inception Cohort, we aimed to identify predictors of clinical response in an international SLE cohort. We also aimed to identify predictors of response in a subset of patients who had a level of disease activity similar to that required for entry into a clinical trial.

## Methods

### SLICC inception Cohort

SLE patients were recruited into the SLICC Inception Cohort from 31 centres across Europe, Asia, North and Central America, from 1999 to 2011. Patients were recruited within 15 months of confirming ≥4 SLE ACR 1997 Updated classification criteria^6^ and assessed at their local centre on an annual basis. Disease activity was recorded using the British Isles Lupus Assessment Group (BILAG) “classic” index^7,8^ and the SLE Disease Activity Index 2000 (SLEDAI-2K)^9^. The BILAG index is the principle scoring system used across the MASTERPLANS consortium studies^10,11^. At each visit, patients also had organ damage assessed using the SLICC/American College of Rheumatology Damage Index(SDI)^12^. In addition, information on therapy, demographic data, co-morbidities and routine laboratory tests were obtained. This study was approved by the University Health Network Research Institute research ethics committee, Toronto, Canada and by the Institutional Research Ethics Boards of all participating centres in accordance with the Declaration of Helsinki’s guidelines for research in humans. All patients provided informed consent.

### Outcomes

Across the MASTERPLANS consortium, 2 outcomes based on the BILAG ”classic” instrument were defined which reflect clinically meaningful reductions in disease activity^11,13^ namely;

1. Major Clinical Response (MCR) was defined using the following criteria at 12 months following the index visit:
  - Reduction in BILAG A and B scores to BILAG C, D or E in all domains
  - Daily prednisolone (or prednisone or equivalent) dose of 7.5mg or less
  - SLEDAI-2K score of 4 or less
2. Improvement was defined using the following criteria at 12 months following the index visit:
  - Reduction in BILAG A or B scores to no more than one BILAG B in previously active organ domains and no new BILAG organ domains (A or B score) involved.
  - Reduced or stable prednisolone (or prednisone or equivalent) use, defined as:
    ⚬ Dose >= 20 mg at recruitment becomes <= 15mg/day
    ⚬ Dose 10 − 20mg at recruitment becomes <= 10mg/day
    ⚬ Dose < 10mg at recruitment dose not increase/day
  - No increase in SLEDAI-2K score

### Patient cohorts studied

From the SLICC Cohort we identified 2 cohorts for analysis:

#### Inception Enrolment Visit cohort

All patients were assessed at their initial baseline visit. We examined the rates and predictors of achieving MCR at 12 months. We did not assess ‘improvement’ in this cohort as many patients did not have sufficiently active disease at cohort entry.

#### ‘Active Disease’ Cohort

We also identified from the whole SLICC cohort patients who had active disease, comparable to that used as entry criteria in many clinical trials. For each patient we identified the first visit at which they had a minimum of one BILAG A or 2 BILAG B scores. This was defined at the index visit in the Active Disease cohort. In this subset, we examined the rates and predictors of achieving MCR and improvement at 12 months following the index visit.

#### Predictors of MCR or improvement

A number of potential predictive factors at the baseline visit were selected on the basis of evidence from other studies that have examined prognostic markers in SLE as well as their availability in the SLICC cohort. Predictors included were:

- Demographics
  ⚬ Gender
  ⚬ Age at SLE diagnosis
  ⚬ Disease duration at baseline
  ⚬ Ethnicity/race
  ⚬ Location
  ⚬ Any post-secondary education (Yes/No)
- Medication at baseline
  ⚬ Oral average prednisolone or equivalent dose (high dose >30mg daily, medium dose 7.5 − 30mg daily, low dose < 7.5mg daily)
  ⚬ Pulse steroid use (Yes/No)
  ⚬ Anti-malarial use (Yes/No)
  ⚬ Immuno-suppressant use (Yes/No)
  ⚬ Individual immunosuppressant and biologics agents)
- Number of A or B scores in BILAG Index, the SLEDAI-2K and SDI scores at baseline
- BILAG score A or B in individual organ systems
- Presence of elevated anti-dsDNA antibodies (as defined by local laboratory parameters)
- Presence of hypocomplementaemia (C3 and/or C4) (as defined by local laboratory parameters)
- Presence of anti-phospholipid antibodies at enrolment^14^
- Comorbidities: hypertension and Diabetes Mellitus
- Lifestyle: alcohol consumption (units per week) and smoking status (current, previous, never)
- SF-36: Mental Component and Physical Component Summary Scores (MCS and PCS)^15^

### Statistical Analysis

Multivariate logistic regressions with shrinkage estimators, i.e., least absolute shrinkage and selection operator (LASSO) and elastic net, were used to build multivariate prediction models^16^. Ten-fold cross-validation with 300 times of repeated random splitting was used; in total 3000 prediction models were built. Each model used a training subsample of the data (9 folds in a specific data split), where the tuning parameters of LASSO and elastic net were selected by cross-validation. Predicted probabilities for the testing samples in the remaining fold were calculated. The predicted probabilities were then averaged across 300 replications (due to repeated random splitting) to generate a final predicted probability for each sample. The prediction performance of the models was summarized by area under Receiver Operating Characteristic (ROC) curves (AUC). We ranked the predictors by their frequencies of being chosen by LASSO among the 3000 models to provide an indication of the importance of the predictors. Additionally, random forests were used to check if there were interactions and nonlinearity among the variables selected by LASSO in more than 50% of the fitted models^17^. Univariate logistic regression models were used to calculate the odd ratios of identified predictors to show the direction and strength of the associations. The analysis was conducted using SAS University edition and R (version 3.6.3).

## Results

We enrolled 1826 patients in the SLICC Inception Cohort that included 1622 (89%) females with a median [IQR] age at diagnosis and disease duration of 32.40 [24.04 − 43.08] years old and 0.40 [0.17 − 0.75] years respectively. A baseline BILAG score was completed in 1492 (81.7%) patients; those with and without a BILAG score had comparable characteristics (Table 1).

### Predictors of Major Clinical Response (MCR) at 12 months in the Inception Enrolment Visit cohort

A total of 1469 patients were analysed of whom 412 (28%) met MCR at 12 months; 103 (7%) who had missing 12-month data could not be classified. Variable selection for factors that may contribute to prediction of MCR was performed using two shrinkage estimators (LASSO and elastic net) and both yielded similar results. Results for LASSO had an Area Under the Curve (AUC) = 0.777. Using the random forest approach with predictors that were selected by LASSO, we found a similar AUC (0.773). Variables selected by LASSO in more than 50% of the prediction models were taken forward into logistic models to individually examine the strength and direction of associations of the chosen predictors.

Predictors of achieving MCR at 12 months (Table 2) included age at diagnosis, residence in Europe, anti-malarial use, SF-36 PCS >=40, alcohol consumption (<= 4 vs. 0 units per week) and smoking. In contrast, African ethnicity, higher baseline disease activity (BILAG or SLEDAI), BILAG A or B scores in musculoskeletal and haematological system, SDI >0, immunosuppressant use with azathioprine or IV cyclophosphamide, and moderate/high oral prednisolone or equivalent doses (>=7.5mg/day at baseline) were inversely associated with achieving MCR at 12-months.

### Predictors of MCR in an ‘Active Disease’ cohort

In total, 924 (63%) of patients had active disease (1 BILAG A or 2 BILAG B’s) at enrolment or one of their follow-up visits; 429 (46.4%) patients in this cohort had this level of disease activity at their enrolment visit. This group included 820 (89%) females and the median [IQR] age at diagnosis was 30.58 [23.18 − 41.34] years old. Patients at the entry visit to this subset had a median disease duration [IQR] of 1.23 [0.29 − 3.45] years (Table 3).

In total, 759 (82%) patients had a 12-month follow-up visit after meeting the ‘Active Disease’ criteria. Of these, 114 (15%) achieved MCR at 12 months; 50 (7%) who had missing data at 12 months were unable to be classified. Results for LASSO had an AUC = 0.732 and using a random forest approach, we found a slightly better AUC (0.757). Variables selected by LASSO in more than 50% of the prediction models were taken forward into logistic models. In this ‘active disease’ cohort, anti-malarial use was associated with MCR at 12 months. Higher disease activity (by BILAG or SLEDAI), hypertension, low complement, active (A or B) haematology domains of BILAG score, higher oral steroid usage and immunosuppressant use were inversely associated with MCR at 12 months (Table 4).

### Predictors of improvement in an Active Disease cohort

Of 759 patients who fulfilled our active disease criteria, 261 (34%) fulfilled our definition of improvement at 12 months; 136 (18%) had missing data and could not be classified. The AUC for different estimators (LASSO and elastic net) and random forest, although very similar had poor prediction accuracy (LASSO: AUC = 0.574; random forest: AUC = 0.645). We examined the selected variables in the univariate logistic models. Factors inversely associated with improvement in the active disease cohort were residence in Mexico, Hispanic and African race/ethnicities, immunosuppressant use, low C3 or C4 and a higher SLEDAI score (Table 5).

Table 6 summarises predictors common to both the inception and active disease cohorts for achieving MCR.

## Discussion

Designing successful clinical trials in SLE remains a major challenge. It is also difficult to develop a precision medicine approach to position such new and existing treatments optimally in the clinic. To date, little is known about predictors of response/non-response to specific agents used to treat SLE^18^. Certain factors (public factors) are not specific to a single agent, rather, they are more markers of likelihood of clinical response in general^19^. Knowledge of such factors is needed to improve stratification/minimisation factors in trials and to improve predictive models for novel SLE therapies.

We assessed predictors of improvement and MCR in a large international lupus inception cohort recruited and managed in their individual centres according to local standards of care. We studied patients at cohort entry and, for the first time, we also identified a subset with active disease of a level similar to that which qualifies for entry to a clinical trial. This latter cohort simulated a trial population and widens the generalisability of our results.

For MCR, in both the inception cohort and the active disease cohort, similar factors predicted MCR and both models performed well with AUC of 0.777 and 0.732 respectively. Several factors identified in the inception enrolment visit group were not seen in the active disease subgroup. This may reflect, in part, limited numbers in the latter analysis (114/759 active versus 412/1469 patients at enrolment). Interestingly, the model for improvement contained similar factors but performed much less well (AUC = 0.574). Our definition of improvement reflects smaller changes in disease activity over time and may be less specific when considering clinical and biological determinants of outcomes compared to the more stringently defined MCR state.

Previous work from our group has also shown differences in outcomes according to race/ethnicity and location in SLE patients^20,21^. In the current analysis, patients of African ancestry were less likely to achieve an MCR response in the inception cohort, and those of both African ancestry and Hispanic race/ethnicity were less likely to achieve improvement in the active disease cohort. This likely reflects the more aggressive disease and adverse clinical outcomes in these populations^19^. Interestingly, SLE patients in Europe were more likely to achieve MCR in the inception enrolment cohort. European location may represent a combination of environmental factors such as reduced exposure to sunlight, infections, environmental pollutants and occupational exposure, all of which have been implicated in the etiology and pathogenesis of SLE^22^. Moreover, the differences in provision of healthcare in the relevant countries in each location may also have influenced disease outcomes. Such differences are important to bear in mind in clinical practice as well as when designing and interpreting clinical trials. A number of trials have observed less marked differences in outcomes in European populations^23–25^ which also may reflect differences in baseline severity of disease and wider use of SOC medications in such patients.

We found a consistent negative association between achieving improvement and MCR in patients with higher disease activity (using both BILAG and SLEDAI). It has been noted in a number of clinical trials that higher disease activity is less likely to elicit a response in the SOC group and our data supports that observation^26^. The non-linear association with SLEDAI in the improvement group also suggests that higher levels of disease activity have a much stronger impact on the inability to achieve improvement with usual therapy. Taken together these observations support the view that clinical trials of novel agents should recruit patients with higher levels of disease activity to provide better discrimination between active novel therapies and usual standard of care^27^. In routine clinical practice, it also emphasises the challenges in getting patients with higher disease activity to low disease activity ‘states’ using conventional therapies.

Patients with pre-existing damage also have a lower likelihood of achieving MCR in the inception cohort. Evidence suggests that patients with higher disease activity are more likely to develop future damage^28,29^. The presence of damage may, also reflect a more severe disease course that is less likely to respond to SOC. Whether damage may also confound the assessment of disease activity in large trials cannot be excluded.

HCQ is the anti-malarial most commonly used in the treatment of SLE and is effective in the reduction of disease flares, steroid dose, organ damage and prevention of thrombotic events^30–33^. Moreover, HCQ has a protective effect on survival^34^. The positive effect of anti-malarial use in disease response is therefore consistent with existing knowledge on the benefits and efficacy of HCQ in SLE. Previous studies have reported adherence rates to HCQ in SLE patients to be low^35–37^ but also that patients recruited to a clinical trial are more likely to comply with medication. Our data supports the benefits of HCQ for controlling disease in a real-world setting and underscores the need to continually support adherence to antimalarials in the clinic. The observed impact of antimalarial usage on clinical response in our study also has important implications for clinical trials. It may be necessary measure HCQ drug levels during trial screening and those with sub-optimal levels could either be excluded or have a ‘run-in’ period and reassessment of disease prior to randomization to ensure a better distinction between the effects of a novel therapy and that of routine SOC.

Our study has a number of limitations. We did not have a validation set in which to confirm our findings. However, we observed important similarities between predictors of MCR in the inception enrolment cohort and in the subset of patients in the active disease cohort. Other studies assessing different but similar ‘states’ such as Lupus Low Disease Activity and remission have found similar factors linked to those outcomes^38–41^, providing some external validation of our findings. The need for further validation is also emphasized by our observations regarding smoking and alcohol consumption being associated with MCR in the inception cohort only. While these may be chance findings, others have found that moderate alcohol consumption may have a protective ‘anti-inflammatory’ in other autoimmune diseases such as rheumatoid arthritis^42^ The classic BILAG index was used in our study due to the availability of long-term classic BILAG data from 1999. Our findings will also need validating using the BILAG-2004 index, albeit the latter is also based on similar concepts to define A and B disease activity. In addition to HCQ, adherence is also an issue with other medications used in SLE and we did not have objective data on drug levels on which to comprehensively assess treatment adherence in this cohort. Finally, we recognise that we were only able to assess disease activity at the next annual review (12-month time-point) and so we will have missed fluctuations in disease activity that may have occurred within the one-year period and before recruitment to the inception cohort. The active disease cohort analysis was however designed to mimic the typical ‘landmark’ analyses in most clinical trials and so does generalize to that situation. Moreover, the 12-month visit was not necessarily when immunosuppressant treatment was changed or steroid dose increased, and dosing was at the discretion of the physician. Patients could have therapies adjusted at any time during the study and we did not analyse whether they were still on the same therapy when response was assessed, as our aim was to assess the phenotype of responders rather than response to specific therapies. Future work will assess if these factors also predict more sustained achievement of such a state over several visits and not just at a single landmark time-point.

We have found a number of baseline factors associated with Improvement and Major Clinical Response over a 12-month period in SLE patients. Race/ethnicity and location all predict overall responses and patients with a higher burden of active disease, pre-existing damage and already taking immunosuppressive therapy were less likely to achieve a Major Clinical Response over 12 months on standard of care. In contrast, antimalarial use predicted better responses. In the clinic these factors help identify patients less likely to respond to standard therapies and provide additional evidence to emphasise ongoing adherence to antimalarials to patients. Such factors are also important to consider as stratification factors, when designing clinical trials or precision medicine studies. Assessing and adjusting these factors when recruiting to clinical trials may help control ‘noise’ in the SOC arm and improve the likelihood of any effective new therapy to have a signal of response.

## Supplementary Material

Supplementary File 1

## Figures and Tables

**Table 1:** Characteristics of inception patient cohort with and without BILAG disease activity assessments available at the enrolment visit

Baseline characteristics	With BILAG	Without BILAG
	N_1_ = 1492	N_2_ = 334
Median age at diagnosis years [Interquartile range (IQR)]	32.52 [24.04 − 43.12]	31.74 [24.09 − 42.78]
Female n(%)	1328 (89)	294 (88)
Ethnicity (%)		
Caucasian	751 (50)	140 (42)
Hispanic	187 (13)	95 (28)
Asian	239 (16)	36 (11)
African	255 (17)	51 (15)
Other	60 (4)	12 (4)
Location (%)		
Canada	346 (23)	72 (22)
United States	425 (28)	114 (34)
Mexico	146 (10)	77 (23)
Europe	419 (28)	58 (17)
Asia	156 (10)	13 (4)
Co-morbidities (%)		
Diabetes, (N_1_ = 579, N_2_ = 128)	16 (3)	4 (3)
Hypertension, (N_1_ = 1452, N_2_ = 326)	561 (39)	112 (34)
Current smokers (%) (N_1_ = 1490, N_2_ = 334)	225 (15)	45 (13)
Alcohol consumption units/week, mean (+/- standard deviation (SD))	1.11 (3.23)	0.60 (1.65)
Post-secondary education (N_1_ = 1413, N_2_ = 307) (%)	886 (63)	178 (58)
**Disease status**		
Disease duration years, median [IQR]	0.39 [0.17 − 0.75]	0.42 [0.16 − 0.73]
Serological markers (%)		
Low C3 and C4, N_1_ = 1385, N_2_ = 297	514 (37)	110 (37)
High anti-dsDNA, N_1_ = 1379, N_2_ = 297	540 (39)	114 (38)
Anti-phospholipid antibodies present (%)*		
Anti-cardiolipin antibodies, N_1_ = 956, N_2_ = 186	121 (13)	17 (9)
Anti-beta2glycoprotein-1, N_1_ = 956, N_2_ = 186	133 (14)	30 (16)
Lupus anticoagulant, N_1_ = 989, N_2_ = 185	199 (20)	42 (23)
SF-36 Physical Component Score, median [IQR], N_1_ = 1263, N_2_ = 253	38.04 [30.74 − 47.54]	39.21 [30.26 − 49.28]
SF-36 Mental Component Score, median [IQR], N_1_ = 1263, N_2_ = 253	45.91 [34.89 − 54.85]	46.80 [35.86 − 54.20]
Total SLEDAI-2K median [IQR], N_1_ = 1488, N_2_ = 330	4 [2 − 8]	4 [2 − 8]
SLICC score		
0	461(31)	112 (34)
1	78 (5)	12 (4)
>=2	45 (3)	9 (3)
NA	907 (61)	201 (60)
**Glucocorticoids**		
Oral prednisolone or equivalent dose mg (current average steroid dose), median, N_1_ = 1026, N_2_ = 237	20 [10 − 30]	22.50 [12.50 − 40]
Pulse IV (%)	73 (5)	12 (4)
**Anti-malarial (%)**	1013 (68)	218 (65)
**Conventional DMARD therapy (%)**		
Azathioprine	239 (16)	66 (20)
Mycophenolate mofetil	108 (7)	36 (11)
Methotrexate	112 (8)	18 (5)
Cyclosporine	23 (2)	1 (0.3)
Cyclophosphamide		
IV	92 (6)	25 (7)
Oral	7 (0.5)	2 (1)
**Biologic DMARD therapy (%)**	14 (1.4)	3 (1.0)

NB: For alcohol consumption median and lower quartile are 0

* Assays performed in Oklahoma Medical Research Foundation Laboratories of the late Dr Morris Reichlin (Dr JT Merrill): Lupus Anticoagulant assay performed using reagents from Rainbow Scientific, Windsor, CT. ELISA assays for anti-cardiolipin and anti-B2GPI used a cut-point as positive as >2SD above the mean of 60 healthy controls^14^

**Table 2:** Univariate odds ratios for predictors of Major Clinical Response (selected by LASSO in 50% of the prediction models) in the Inception Enrolment Visit cohort

Predictors	Odds Ratio	95% confidence interval
Age at diagnosis (one-year increase from 35 years)	1.021	1.012 − 1.029
Residence in Europe (vs. Canada)	1.581	1.157 − 2.160
African race/ethnicity (vs. Caucasian)	0.385	0.264 − 0.560
Alcohol consumption (<=4 units per week vs. 0 or not available)	1.733	1.307 − 2.299
Current smokers	1.448	1.056 − 1.986
Number of BILAG A or B system scores (1 vs. 0)	0.465	0.346 − 0.627
Number of BILAG A or B system scores (>=2 vs. 0)	0.161	0.106 - 0.245
Musculoskeletal BILAG score (A or B vs. C, D or E)	0.378	0.248 − 0.574
Haematological BILAG score (A or B vs. C, D or E)	0.324	0.220 − 0.478
SLEDAI score (Increase by 1-point)	0.831	0.803 − 0.860
SLICC Damage Index (SDI) score (1 vs. 0)	0.401	0.244 − 0.660
SF-36 PCS score (>=40 vs. <40)	1.750	1.358 − 2.254
Immunosuppressant use	0.430	0.335 − 0.551
Azathioprine use	0.470	0.333 − 0.663
IV cyclophosphamide use	0.206	0.099 − 0.429
Anti-malarial use	2.392	1.818 − 3.146
Oral prednisolone or equivalent dose (high) (>30 mg/day vs. <7.5mg/day)	0.183	0.105 − 0.317
Oral prednisolone or equivalent dose (moderate) (7.5 − 30 mg/day vs. <7.5mg/day)	0.500	0.337 − 0.742

**Table 3:** Characteristics of the Active Disease cohort at the first visit where the patients satisfied the active disease criteria (at least 1 A or 2 B in BILAG scores).

Baseline characteristics	Total cohort n = 924
Median age at diagnosis years [Interquartile range (IQR)], n = 923	30.58 [23.18 − 41.34]
Female n(%)	820 (89)
Ethnicity (%)	
Caucasian	385 (42)
Hispanic	179 (19)
Asian	152 (16)
African	174 (19)
Other	34 (4)
Location (%)	
Canada	218 (24)
United States	237 (26)
Mexico	156 (17)
Europe	213 (23)
Asia	100 (11)
Co-morbidities (%)	
Diabetes, n = 607	16 (3)
Hypertension, n = 900	375 (42)
Current smokers (%), n = 922	134 (15)
Alcohol consumption units/week*, mean (standard deviation (SD)), n = 914	0.76 (2.11)
Post-secondary education (%), n = 863	502 (58)
**Disease status**	
Disease duration years, median [IQR]	1.23 [0.29 − 3.45]
Serological markers (%)	
Low C3 and C4, n = 879	399 (45)
High anti-dsDNA, n = 875	420 (48)
Anti-phospholipid antibodies present (%)**	
Anti-cardiolipin antibodies, n = 430	63 (15)
Anti-beta2glycoprotein-1, n = 431	65 (15)
Lupus anticoagulant, n = 451	100 (22)
SF-36 Physical Component Score, median [IQR], n = 743	37.45 [28.94 − 47.17]
SF-36 Mental Component Score, median [IQR], n = 734	44.98 [34.51 − 53.89]
Total SLEDAI-2K median [IQR], n = 920	7 [4 − 12]
SLICC score	
0	369 (40)
1	124 (13)
2	60 (6)
3	39 (4)
>= 4	17 (2)
NA	315 (34)
Classic BILAG, A or B scores (%)	
Constitutional	159 (17)
Mucocutaneous	317 (34)
Neuro-psychiatric	58 (6)
Musculoskeletal	335 (36)
Cardio-respiratory	46 (5)
Vasculitis	87 (9)
Renal	480 (52)
Haematological	406 (44)
**Glucocorticoids**	
Average oral prednisolone or equivalent dose mg*, median, n = 804	11.55 [5 − 27.7]
Pulse IV (%)	61 (7)
**Anti-malarial (%)**	603 (65)
**Conventional DMARD therapy (%), n = 921**	
Azathioprine	232 (25)
Mycophenolate mofetil	131 (14)
Methotrexate	93 (10)
Cyclosporin	20 (2)
Cyclophosphamide	
IV	86 (9)
Oral	11 (1)
Other	27 (3)
**Biologic DMARD therapy (%), n = 921**	
Rituximab	12 (1)
Belimumab	4 (0.4)
Abatacept	3 (0.3)
Other	10 (1)

NB: For alcohol consumption median, lower and upper quartile are all 0.

* For oral prednisolone or equivalent dose, if study entry-criteria met at enrolment then average prednisolone or equivalent dose for the current course is stated and if study entry-criteria met at a follow-up visit then average prednisolone or equivalent dose since the last visit is stated.

** 8 Assays performed in Oklahoma Medical Research Foundation Laboratories of the late Dr Morris Reichlin (Dr JT Merrill): Lupus Anticoagulant assay performed using reagents from Rainbow Scientific, Windsor, CT. ELISA assays for anti-cardiolipin and anti-B2GPI used a cut-point as positive as >2SD above the mean of 60 healthy controls^14^

**Table 4:** Univariate odds ratios for predictors of Major Clinical Response (selected by LASSO in 50% of the prediction models) in the Active Disease cohort

Predictors	Odds Ratio	95% Confidence Interval
Anti-malarial use	2.273	1.406 − 3.676
Hypertension	0.573	0.371 − 0.884
Low C3 or C4	0.481	0.310 − 0.746
Number of BILAG A or B system scores (>=2 vs. 1)	0.439	0.288 − 0.668
Haematology (BILAG) score (A or B vs. C, D or E)	0.617	0.404 − 0.941
SLEDAI score (Increase by 1-point)	0.884	0.844 − 0.926
Oral prednisolone or equivalent dose (Moderate (>7.5 -30mg/day) vs. low (<= 7.5mg/day))	0.327	0.174 − 0.617
Immunosuppressant use	0.441	0.292 − 0.668

**Table 5:** Univariate odds ratios for predictors of improvement (selected by LASSO in 50% of the prediction models) in the Active Disease cohort

Predictors	Odds Ratio	95% Confidence Interval
Residence in Mexico (vs. Canada)	0.473	0.285 − 0.785
Hispanic race/ethnicity (vs. Caucasian)	0.381	0.244 − 0.595
African race/ethnicity (vs. Caucasian)	0.543	0.338 − 0.841
Low C3 or C4	0.602	0.432 − 0.839
SLEDAI (linear)	0.993	0.824-1.197
SLEDAI(quadratic)	0.852	0.737-0.986
Immunosuppressant use	0.607	0.439 − 0.838

**Table 6:** Summary of predictors associated with a lower or higher probability of achieving MCR in both the Inception Enrolment cohort and the Active Disease cohorts

**Higher probability of achieving MCR in Inception Enrolment and Active Disease cohort**
Anti-malarial use
**Lower probability of achieving MCR in Inception Enrolment and Active Disease cohort**
Number of BILAG A or B system scores (>=2 vs. 1 for active disease cohort, >=2 vs. 0 and 1 vs 0 for inception cohort)
Haematology BILAG score (A or B vs. C, D or E)
SLEDAI score (per unit increase)
Immunosuppressant use
Oral prednisolone or equivalent dose (Moderate (>7.5 -30mg/day) vs. low (<= 7.5mg/day))
**Lower probability of achieving MCR in Inception Enrolment and Active Disease cohort and improvement in Active Disease cohort**
SLEDAI (per unit increase)
Immunosuppressant use

## Lay summary

Little is known about factors that predict which patients with Systemic Lupus Erythematosus (SLE) respond well to usual treatments; such information is important when we design studies of new treatments for SLE. We used data from a large international SLE patient population to identify factors associated with a significant reduction in disease activity, regardless of treatment administered. Patients were divided into two groups: an ‘active disease’ group, where patients had disease activity similar to that usually required for entry into clinical trials, and an ‘inception enrolment visit’ group, where patients were assessed regardless of their initial level of disease activity.

We assessed patients who had a response (improvement) or a major response at 12 months. A total of 1492 patients were studied in the inception enrolment visit group and 924 in the ‘active disease’ group. We found a number of factors associated with a major response in both the inception enrolment and the active disease groups. A higher probability of major response was associated with antimalarial use. A lower probability was seen in patients with higher overall disease activity as well as those where their blood cells were affected by SLE. Patients were also less likely to have a major response if they were already taking higher doses of steroids or taking immunosuppressive drugs at baseline.

All of these factors are important to consider when patients are being assessed in clinic as they help identify patients more or less likely to respond to standard therapies. When designing clinical trials in SLE these factors also need to be balanced between the groups to improve the chances that a new drug might be shown to be effective.

## References

[1] Cervera R, Khamashta MA, Font J (2003). Morbidity and mortality in systemic lupus erythematosus during a 10-year period: a comparison of early and late manifestations in a cohort of 1,000 patients. Medicine (Baltimore).

[2] Borchers AT, Keen CL, Shoenfeld Y, Gershwin ME (2004). Surviving the butterfly and the wolf: mortality trends in systemic lupus erythematosus. Autoimmun Rev.

[3] Carter EE, Barr SG, Clarke AE (2016). The global burden of SLE: prevalence, health disparities and socioeconomic impact. Nat Rev Rheumatol.

[4] Tektonidou MG, Lewandowski LB, Hu J, Dasgupta A, Ward MM (2017). Survival in adults and children with systemic lupus erythematosus: a systematic review and Bayesian meta-analysis of studies from 1950 to 2016. Ann Rheum Dis.

[5] Ruiz-Irastorza G, Bertsias G (2020). Treating systemic lupus erythematosus in the 21st century: new drugs and new perspectives on old drugs. Rheumatology.

[6] Hochberg MC (1997). Updating the American college of rheumatology revised criteria for the classification of systemic lupus erythematosus. Arthritis Rheum.

[7] Hay EM, Bacon PA, Gordon C (1993). The BILAG index: a reliable and valid instrument for measuring clinical disease activity in systemic lupus erythematosus. QJM An Int J Med.

[8] Isenberg DA, Gordon C, BILAG Group (2000). British Isles Lupus Assessment Group. From BILAG to BLIPS--disease activity assessment in lupus past, present and future. Lupus.

[9] Gladman DD, Ibañez D, Urowitz MB (2002). Systemic lupus erythematosus disease activity index 2000. J Rheumatol.

[10] Murphy C-L, Yee C-S, Gordon C, Isenberg D (2016). From BILAG to BILAG-based combined lupus assessment-30 years on. Rheumatology (Oxford).

[11] Davies JC, Carlsson E, Midgley A (2021). A panel of urinary proteins predicts active lupus nephritis and response to rituximab treatment. Rheumatology (Oxford).

[12] Gladman D, Ginzler E, Goldsmith C (1996). The development and initial validation of the systemic lupus international collaborating clinics/American college of rheumatology damage index for systemic lupus erythematosus. Arthritis Rheum.

[13] Merrill JT, Neuwelt CM, Wallace DJ (2010). Efficacy and safety of rituximab in moderately-to-severely active systemic lupus erythematosus: the randomized, double-blind, phase II/III systemic lupus erythematosus evaluation of rituximab trial. Arthritis Rheum.

[14] Hanly JG, Li Q, Su L (2018). Cerebrovascular Events in Systemic Lupus Erythematosus: Results From an International Inception Cohort Study. Arthritis Care Res (Hoboken).

[15] Ware J, Kosinski M (2001). SF-36 physical & mental health summary scales: a manual for users of version 1. https://scholar.google.com/scholar?cluster=12170310011220991776&hl=en&as_sdt=2005&sciodt=0,5.

[16] Tibshirani R (1996). Regression Shrinkage and Selection via the lasso. J R Stat Soc B.

[17] Hastie T, Tibshirani R, Friedman JH (2006). The elements of statistical learning: data mining, inference, and prediction.

[18] Pirone C, Mendoza-Pinto C, van der Windt DA, Parker OB, Sullivan M, Bruce IN (2017). Predictive and prognostic factors influencing outcomes of rituximab therapy in systemic lupus erythematosus (SLE): A systematic review. Semin Arthritis Rheum.

[19] Dall’Era M, Bruce IN, Gordon C, Manzi S, McCaffrey J, Lipsky PE (2019). Current challenges in the development of new treatments for lupus. Ann Rheum Dis.

[20] Parker B, Urowitz MB, Gladman DD (2015). Impact of early disease factors on metabolic syndrome in systemic lupus erythematosus: data from an international inception cohort. Ann Rheum Dis.

[21] Bruce IN, O’Keeffe AG, Farewell V (2015). Factors associated with damage accrual in patients with systemic lupus erythematosus: results from the Systemic Lupus International Collaborating Clinics (SLICC) Inception Cohort. Ann Rheum Dis.

[22] Parks CG, de Souza Espindola Santos A, Barbhaiya M, Costenbader KH (2017). Understanding the role of environmental factors in the development of systemic lupus erythematosus. Best Pract Res Clin Rheumatol.

[23] Houssiau FA, D’Cruz D, Sangle S (2010). Azathioprine versus mycophenolate mofetil for long-term immunosuppression in lupus nephritis: results from the MAINTAIN Nephritis Trial. Ann Rheum Dis.

[24] Tesar V, Hruskova Z (2015). Lupus Nephritis: A Different Disease in European Patients?. Kidney Dis (Basel, Switzerland).

[25] Furie R, Rovin BH, Houssiau F (2020). Two-Year, Randomized, Controlled Trial of Belimumab in Lupus Nephritis. N Engl J Med.

[26] Merrill JT, Shanahan WR, Scheinberg M, Kalunian KC, Wofsy D, Martin RS (2018). Phase III trial results with blisibimod, a selective inhibitor of B-cell activating factor, in subjects with systemic lupus erythematosus (SLE): results from a randomised, double-blind, placebo-controlled trial. Ann Rheum Dis.

[27] Merrill JT, Manzi S, Aranow C (2018). Lupus community panel proposals for optimising clinical trials: 2018. Lupus Sci Med.

[28] Gladman DD, Urowitz MB (1999). The SLICC/ACR damage index: progress report and experience in the field. Lupus.

[29] Stoll T, Sutcliffe N, Mach J, Klaghofer R, Isenberg DA (2004). Analysis of the relationship between disease activity and damage in patients with systemic lupus erythematosus-a 5-yr prospective study. Rheumatology.

[30] Fessler BJ, Alarcón GS, McGwin G (2005). Systemic lupus erythematosus in three ethnic groups: XVI. Association of hydroxychloroquine use with reduced risk of damage accrual. Arthritis Rheum.

[31] Hironari Hanaoka HL Hydroxychloroquine Improves Disease Activity and Allows Reduction of Corticosteroid Dose Regardless of Background Treatment in Japanese Patients with Systemic Lupus Erythematosus - ACR Meeting Abstracts. https://acrabstracts.org/abstract/hydroxychloroquine-improves-disease-activity-and-allows-reduction-of-corticosteroid-dose-regardless-of-background-treatment-in-japanese-patients-with-systemic-lupus-erythematosus/.

[32] Aouhab Z, Hong H, Felicelli C, Tarplin S, Ostrowski RA (2019). Outcomes of Systemic Lupus Erythematosus in Patients who Discontinue Hydroxychloroquine. ACR open Rheumatol.

[33] Konig Maximilian, Jessica Li MP Hydroxychloroquine Blood Levels and Risk of Thrombotic Events in Systemic Lupus Erythematous - ACR Meeting Abstracts. https://acrabstracts.org/abstract/hydroxychloroquine-blood-levels-and-risk-ofthrombotic-events-in-systemic-lupus-erythematous/.

[34] Alarcón GS, McGwin G, Bertoli AM (2007). Effect of hydroxychloroquine on the survival of patients with systemic lupus erythematosus: data from LUMINA, a multiethnic US cohort (LUMINA L). Ann Rheum Dis.

[35] Kweon Seong-Min ACR (2017). Compliance and Persistence with Hydroxychloroquine in Patients with Systemic Lupus Erythematosus - ACR Meeting Abstracts. https://acrabstracts.org/abstract/compliance-and-persistence-with-hydroxychloroquine-in-patients-with-systemic-lupus-erythematosus/.

[36] Costedoat-Chalumeau N, Houssiau F, Izmirly P (2018). A Prospective International Study on Adherence to Treatment in 305 Patients With Flaring SLE: Assessment by Drug Levels and Self-Administered Questionnaires. Clin Pharmacol Ther.

[37] Liu LH, Fevrier HB, Goldfien R, Hemmerling A, Herrinton LJ (2019). Understanding Nonadherence with Hydroxychloroquine Therapy in Systemic Lupus Erythematosus. J Rheumatol.

[38] Golder V, Kandane-Rathnayake R, Hoi AY-B (2016). Frequency and predictors of the lupus low disease activity state in a multi-national and multi-ethnic cohort. Arthritis Res Ther.

[39] Tani C, Vagelli R, Stagnaro C, Carli L, Mosca M (2018). Remission and low disease activity in systemic lupus erythematosus: an achievable goal even with fewer steroids? Real-life data from a monocentric cohort. Lupus Sci Med.

[40] Ugarte-Gil MF, Wojdyla D, Pons-Estel GJ (2019). Predictors of Remission and Low Disease Activity State in Systemic Lupus Erythematosus: Data from a Multiethnic, Multinational Latin American Cohort. J Rheumatol.

[41] Babaoglu H, Li J, Goldman D, Magder LS, Petri M (2019). Predictors of predominant Lupus Low Disease Activity State (LLDAS-50). Lupus.

[42] Kallberg H, Jacobsen S, Bengtsson C (2009). Alcohol consumption is associated with decreased risk of rheumatoid arthritis: results from two Scandinavian case-control studies. Ann Rheum Dis.

